# Supplementary material for: Spatiotemporal Variation and Predictors of Unsuppressed Viral Load among HIV-Positive Men and Women in Rural and Peri-Urban KwaZulu-Natal, South Africa
Source: Trop Med Infect Dis. 2022 Sep 6;7(9):232. doi: 10.3390/tropicalmed7090232 (PMC9502339; doi:10.3390/tropicalmed7090232)
Supplement: Supplementary file 1 [file tropicalmed-07-00232-s001.zip › Supplementary files/supplementary file 1/Revised CLEAN Supplementary Materials 15 August 2022 .pdf]

Spatiotemporal Variation and Predictors of Unsuppressed Viral Load Among HIV Positive Men and Women in Rural and Peri-urban KwaZulu-Natal, South Africa

Authors

Adenike O Soogun [1,2,*,†], Ayesha BM Kharsany [2,3,#], Temesgen Zewotir [1], Delia North [1], Ebenezer Ogunsakin [4] and Perry Rakgoale [5]

† Adenike O Soogun and Ayesha BM Kharsany are Joint first authors.

Supplementary tables and figures

Supplementary Table S1: Characteristics of enrolled HIV positive men and women with viral load measurement in Vulindlela (rural) and Greater Edendale (peri-urban) area in uMgungundlovu district, KwaZulu-Natal province, South Africa**.**

Supplementary Table S2: Bivariate Survey logistics regression model examining predictors of unsuppressed viral load among HIV positive men and women in Vulindlela (rural) and Greater Edendale (peri-urban) area in uMgungundlovu district, KwaZulu-Natal Province, South Africa.

Supplementary Table S2: Values of model comparison measures, DIC.

Supplementary Figure S1: Potential predictors of HIV unsuppressed viral load.

Supplementary Figure S2: Prevalence of composite HIV viral load <400 copies/ml (suppressed) and ≥400 copies/ml (unsuppressed by gender and overall, among HIV positive men and women. **(B)** Progress towards 95-95-95 UNAIDS target, overall and by gender. First 95: Proportion of HIV positive individual who knew their status; Second 95: Proportion of HIV positive individuals who knew their status and self-reported to be on ART; Third 95: Proportion of HIV positive individuals who knew their status, self-reported to be on ART and had HIV viral load < 400 copies/ml.

Table S1: Characteristics of enrolled HIV positive men and women with viral load measurement in Vulindlela (rural) and Greater Edendale (peri-urban) area in the uMgungundlovu district, KwaZulu-Natal province, South Africa

Characteristics	Study sample (N= 7824) n (%)
**Socio-demographic**	
Median age [IQR]	33 (27-39)
Median household size [IQR]	4 (2-6)
Study year	
2014 survey	3956 (50.6%)
2015 survey	3868 (49.4%)
Gender	
15-19	337 (4.3%)
20-24	929 (12.1%)
25-29	1484 (18.7%)
30-34	1669 (21.3%)
35-39	1415 (19.1%)
40-44	1211 (15.1%)
45-49	779 (9.4%)
Gender	
Women	5893 (64.5%)
Men	1931 (35.5%)
Marital status	
Never married	6479 (83.8%)
Ever married	1345 (16.2%)
Education level	
Incomplete High School	4366 (57.6%)
Complete High School	3231 (40.3%)
No Schooling	227 (2.1%)
Duration in the community	
Always	5088 (66.8%)
Moved in less than 1 year ago	266 (3.0%)
Moved in more than 1 year ago	2470 (30.2%)
Away from home for >1 month	
Yes	714 (9.3%)
No	7110 (90.7%)
Run out of money last 12 months [A]	
Yes	2917 (38.9%)
No	4893 (61.1%)
Had meal cut last 12 months [C]	
Yes	2598 (34.9%)
No	5212 (65.1%)
Accessing health care [D]	
Yes	4395 (58.3%)
No	3415 (41.7%)
Monthly household income [E]	
>R2 500	1836 (26.6%)
≤R2 500	5039 (63.0%)
No income	939(10.4%)
Place of residence	
Rural	2606 (46.8%)
Peri-urban	5222 (53.2%)
**Behavioral**	
Median number of sex partner last 12 months [IQR]	1 [1-2]
Median number of lifetime sex partner [IQR]	3 [2-5]
Had sex in last 12 months	
Yes	6306 (81.4%)
No	1518 (18.6%)
Forced sex first time	

Yes	182 (2.1%)
No	7552 (96.6%)
Don't remember	90(1.3%)
Alcohol consumption	
Yes	1928 (27.8%)
No	5896 (72.2%)
**HIV perception and knowledge**	
Number of lifetime HIV test (median [IQR]	2 [1-3]
Ever tested for HIV	
Yes	6959 (89.6%)
No	865 (10.4%)
Perceived risk of contracting HIV	
Likely to acquire HIV	1476 (19.9%)
Not likely to acquire HIV	1571 (19.8%)
Already infected	4777 (60.3%)
**Medical history**	
Ever tested for TB	
Yes	3862 (52.1%)
No	3962 (47.9%)
Exposed to TB last 12 months	
Yes	364 (5.0%)
No	7442 (94.9%)
Don't want to disclose	18 (0.1%)
Ever diagnosed with TB	
Yes	959 (14.1%)
No	6865 (85.9%)
On medication to prevent TB	
Yes	808 (10.4%)
No	7016 (89.6%)
Ever had any STI symptoms	
Yes	306 (3.3%)
No	7518 (96.7%)
Ever diagnosed with STI	
Yes	743 (10.7%)
No	7081 (89.3%)
**Biological**	
On ART	
Yes	3942 (50.8%)
No	3882 (49.2)
ARV dosage [F]	
Fixed/single dose	3334 (87.2%)
Multiple	481 (12.7%)
No response	127 (0.03%)
**CD4 cell count** [G]	
< 350 cells/µL	2097 (27.7%)
350 – 499 cells/µL	1697 (22.1%)
≥500 cells/µL	3990 (49.7%)

Missing data for; [A, B, C, D, =]14 ; [E] 10 , [F] 127 , [G] 40 ; IQR = Interquartile range .
Missing data excluded from percentage calculation. ZAR= South African Rands (ZAR 15 ~US$1)
Ever had any STI symptoms = any symptoms of abnormal vaginal discharge, burning or pain when passing urine, or presence of any genital ulcers/warts

Table S2: Bivariate Survey logistics regression model examining predictors of unsuppressed HIV viral load among HIV positive men and women in Vulindlela (rural) and Greater Edendale (peri-urban) area in the uMgungundlovu district, KwaZulu-Natal province, South Africa

Characteristics	**Unadjusted OR (95% CI)**	**P-value**
**Socio-demographic**		
**Year (ref: 2014)**		
2015	0.70 (0.63 – 0.78)	<0.0001*
**Gender (ref: female)**		
Male	1.66 (1.48 - 1.85)	<0.0001*
**Age group (ref: 15-19)**		
20-24	1.16 (0.85 - 1.59)	0.352
25-29	0.67 (0.51 - 0.89)	0.005*
30-34	0.46 (0.35 -0.60)	<0.0001*
35-39	0.31 (0.23 -0.41	<0.0001*
40-44	0.30 (0.23 -0.41	<0.0001*
45-49	0.25 (0.18 - 0.34)	<0.0001*
**Education level (Ref: Complete high schooling)**		
Incomplete High schooling	0.81 (0.72 - 0.90)	<0.0001*
**Away from home more than 1 month (Ref: No)**		
Yes	1.41 (1.17 - 1.70)	<0.0001*
**Community duration (ref: Always)**		
Moved here less than 1 year ago	1.07 (1.43 -0.66)	0.664
Moved here more than 1 year ago	0.77 (0.69 - 0.87)	<0.0001*
**Marital Status (ref: Ever married)**		
Never married	1.63 (1.38 - 1.92)	<0.0001*
**Enumeration area (Ref: Rural)**		
Peri-urban	1.08 (0.96 - 1.23)	0.206
**Ran out of money last 12 months (Ref: No)**		
Yes	0.88 (0.79 - 0.98)	0.026*
**Had meal cut last 12 months (Ref: No)**		
Yes	0.87 (0.78 - 0.98)	0.024*
**Accessing health care (Ref: Yes)**		
No	1.66 (1.48 - 1.86)	<0.0001*
**Income (Ref: >R2500)**		
≤ R2500	1.08 (0.96 - 1.21)	0.190
No income	1.25 (1.04 - 1.49)	0.018
**Sexual behavioral**		
**Had sex in last 12 months (Ref: Yes)**		
No	0.89 (0.78 - 1.01)	0.077
**Number of sex partner last 12 months (Ref: 0 partner)**		
1 partner	1.01 (0.89 - 1.15)	0.838
2 or More partners	1.56 (1.26 -1.92)	<0.0001*
**Number of lifetime sex partner (Ref: 0-1 partner)**		
2 or more partners	0.75 (0.64 - 0.89)	< 0.0001*
**Forced sex first time (Ref: No)**		
Yes	0.89 (0.64 - 1.23)	0.471
Don't remember	1.53 (1.23 - 1.91)	<0.0001*
**Alcohol consumption (Ref: No)**		
Yes	1.78 (1.58 - 2.00)	<0.0001*
**HIV perception and testing knowledge**		

**Ever tested for HIV (Ref: Yes)**		
No	3.54 (2.84 - 4.42)	<0.0001*
**Number of lifetime HIV test (Ref: 2 or more times)**		
1 time	0.84 (0.74 - 0.95)	0.005*
Never	3.36 (2.68 - 4.20)	<0.0001*
**Knowledge of HIV status (Ref: Positive)**		
Negative	6.28 (5.52 - 7.15)	<0.0001*
**Perceived risk of contracting HIV (Ref: Already infected)**		
Likely to Acquire HIV	5.27 (4.51 - 6.16)	<0.0001*
Not likely to Acquire HIV	6.36 (5.45 - 7.43)	<0.0001*
**Medical history**		
**Ever tested for TB (Ref: No)**		
Yes	0.34 (0.31 - 0.38)	<0.0001*
**Exposed to TB last 12 months (Ref: No)**		
Yes	0.78 (0.61 - 1.00)	0.053*
**Ever Diagnosed with TB (Ref: No)**		
Yes	0.43 (0.36 - 0.51)	<0.0001*
**On TB medication (Ref: No)**		
Yes	0.30 (0.24 - 0.36)	<0.0001*
**Ever had any STI symptoms (Ref: No)**		
Yes	0.92 (0.71 - 1.20)	0.533
**Ever diagnosed with STI (Ref: No)**		
Yes	1.18 (0.99 - 1.42)	0.070
**Biological**		
**On ARV (Ref: Yes)**		
No	16.57 (14.43 - 19.02)	< 0.0001*
**ARV dosage (Ref: Fixed dosage)**		
Multiple dosage	2.72 (2.10 - 3.52)	<0.0001*
**CD4 count category (Ref: <350 cells/μL)**		
350-499 cells/μL	0.40 (0.34 - 0.47)	<0.0001*
≥500 cells/μL	0.21 (0.18 - 0.24)	<0.0001*

*Significant at 5% level of significance. IQR = Interquartile range. OR= Odds Ratio. CI= Confidence Interval ZAR= South African Rands (ZAR 15 ~US$1). Ever had any STI symptoms = any symptoms of abnormal vaginal discharge burning or pain when passing urine, or presence of any genital ulcers/warts

Table S3. Values of model comparison measures, DIC.

Parameters	GAM	Structured	Unstructured
DIC	6410.15	5903.19	5894.51
**pD**	63.93	77.69	81.45

DIC: Deviance Information Criteria. pD: effective numbers of parameters.

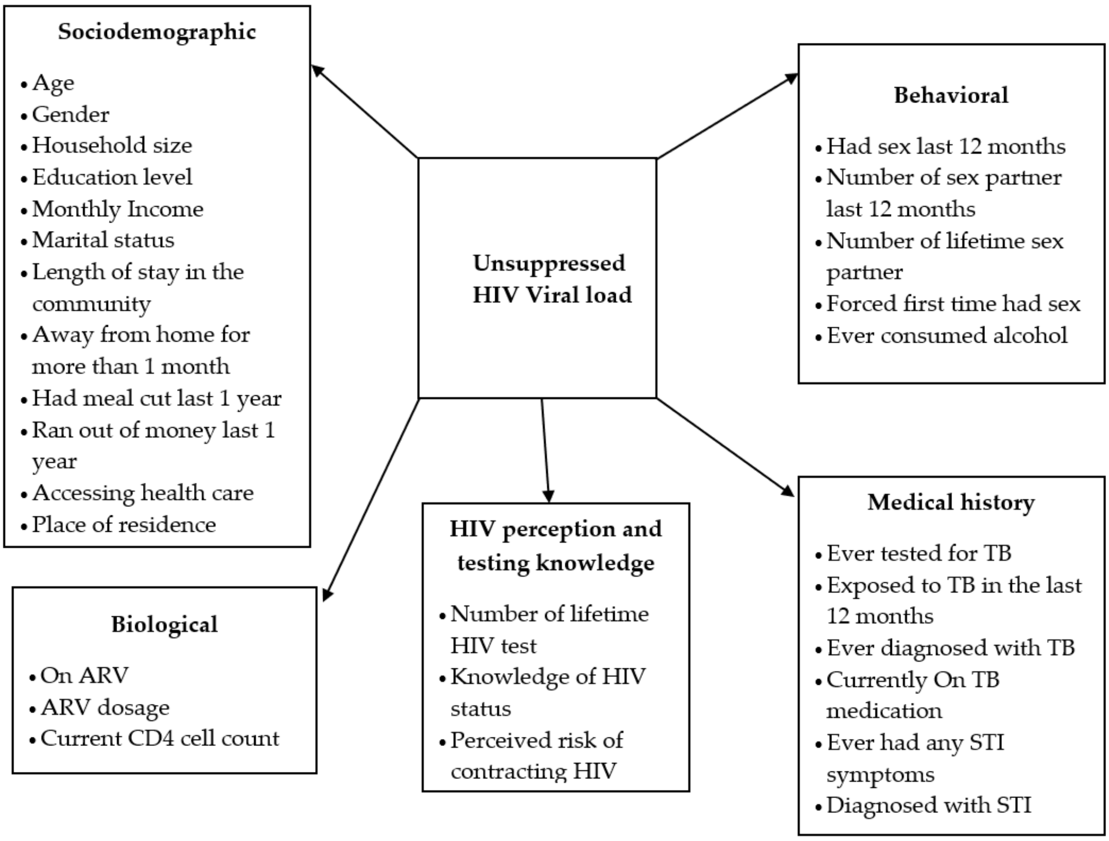

Figure S1: Potential predictors of unsuppressed HIV viral load

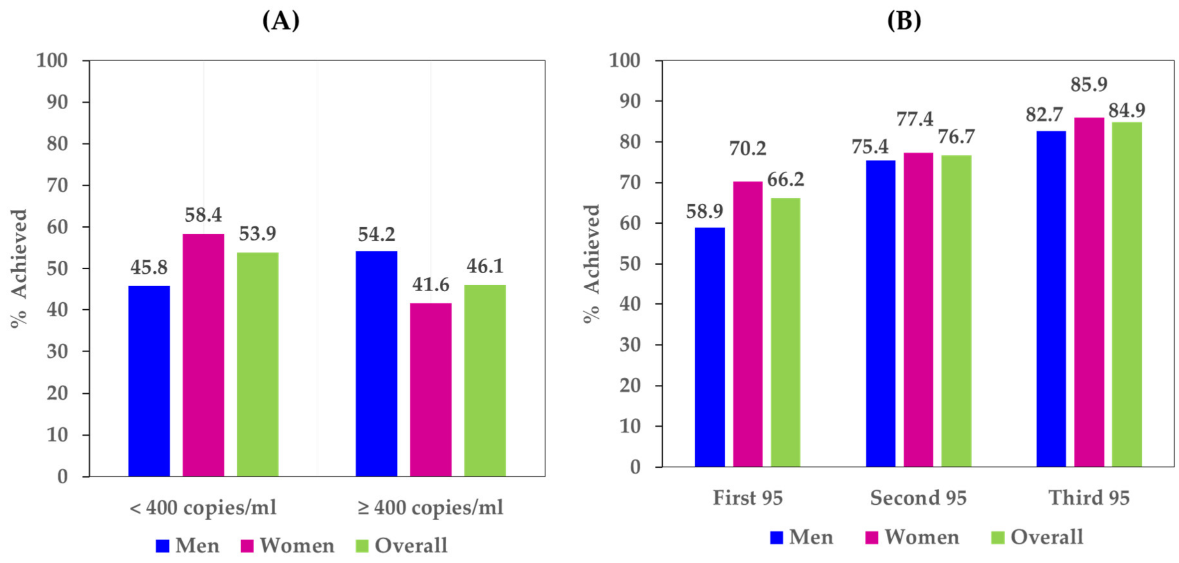

Figure S2: (A) Prevalence of composite HIV viral load <400 copies/ml (suppressed) and ≥400 copies/ml (unsuppressed by gender and overall, among HIV positive men and women. **(B)** Progress towards UNAIDS 95-95-95 targets by gender and overall. First 95: Proportion of HIV positive individuals who knew their status. Second 95: Proportion of HIV positive individuals who knew their status and self-reported to be on ART; Third 95: Proportion of HIV positive individuals who knew their status, self-reported to be on ART and had HIV viral load < 400 copies/ml.
